# Child sexual abuse and sleep disturbances among adolescents: the role of post-traumatic stress disorder symptoms

**DOI:** 10.3389/fpsyg.2025.1580679

**Published:** 2025-08-21

**Authors:** Martine Hébert, Valérie Théorêt, Amélie Tremblay-Perreault, Antonio Zadra

**Affiliations:** 1 Département de Sexologie, Université du Québec à Montréal, Montréal, QC, Canada; 2 École de Criminologie, Université de Montréal, Montréal, QC, Canada; 3 Département de Psychologie, Université de Montréal, Montréal, QC, Canada

**Keywords:** trauma, sexual violence, adolescents, sleep impairment, sexual abuse

## Abstract

**Introduction:**

Sleep disturbances represent a major concern for many adolescents. While adolescents with a history of trauma may be particularly vulnerable to sleep disturbances, the mechanisms underlying the association between childhood sexual abuse and sleep disturbances (e.g., having trouble falling asleep, nightmares) remain understudied. The present study aimed to: (1) explore sleep disturbances among adolescents with a history of childhood sexual abuse, and (2) investigate the mediating role of PTSD symptoms in the association between childhood sexual abuse and sleep disturbances.

**Methods:**

Data were drawn from a representative sample of 8,194 adolescents in grades 9 to 11. The history of childhood sexual abuse and PTSD symptoms were assessed at Time 1, whereas sleep disturbances were assessed 6 months later (Time 2) using self-reported questionnaires.

**Results:**

Adolescents who experienced childhood sexual abuse were more likely to report sleep disturbances than those who experienced other forms of childhood adversity. Sexual abuse was associated with higher levels of PTSD symptoms at Time 1, which, in turn, predicted more sleep disturbances at Time 2.

**Discussion:**

These findings suggest that sleep disturbances should be monitored and addressed in trauma-focused therapy for youth victims of childhood sexual abuse.

## 1 Introduction

The importance of sleep in adolescence is well established, as it supports brain development, cognitive function, and mental well-being (Anastasiades et al., 2022; Spruyt, 2024; Tarokh et al., 2016). Many concerns have been expressed and documented regarding deficient sleep in adolescents. Factors such as natural changes in the circadian rhythm, school start times, and evening use of social media have all been linked to sleep problems in adolescents (Barlaan et al., 2022; Owens and Weiss, 2017; Tarokh et al., 2016). Trauma exposure in adolescence has also been found to have both immediate and long-lasting impacts on sleep (Barrera-Valencia et al., 2025; Giannakopoulos and Kolaitis, 2021; Zarchev et al., 2025). In their review, Langevin et al. (2022) reported that child sexual abuse (CSA) is associated with various sleep disturbances during adolescence, including lower levels of sleep efficiency, sleep satisfaction, and sleep duration. However, few studies have attempted to identify mechanisms explaining the association between exposure to trauma and sleep disturbances in youth, and even fewer have specifically focused on a sample of CSA victims. Given that CSA sometimes takes place in sleep-related contexts, CSA may have a particularly strong relationship with sleep disturbances when compared to other trauma types. Moreover, CSA appears to be the most synergistic form of trauma exposure, implying that having experienced CSA potentiates its consequences when paired with another adverse childhood experience (Briggs et al., 2021).

Among various mechanisms linking CSA to sleep disturbances, symptoms of post-traumatic stress disorder (PTSD) are of interest. PTSD is a well-known sequela of CSA (Hailes et al., 2019), and PTSD and sleep problems are so closely related that nightmares and other sleep disturbances are included as symptoms of intrusion and alterations in arousal in the DSM-5 criteria for PTSD (American Psychiatric Association, 2022). The hyperarousal-based theory of sleep disturbances posits that PTSD induces a state of heightened arousal that disrupts the conditions necessary for good sleep quality (Mellman, 1997). Additionally, PTSD can lead survivors to reexperience trauma-related cues in their minds, triggering physiological arousal and negative emotions, which further interfere with sleep (Giannakopoulos and Kolaitis, 2021). It should be noted, however, that sleep problems are not only a symptom of past trauma, but also a potential mechanism through which trauma contributes to long-term health and psychological difficulties (Anda et al., 2006; Chapman et al., 2011; Hillebrant-Openshaw and Wong, 2023). Several studies have supported PTSD as a predictor of sleep disturbances, although the vast majority of these have been conducted on adult participants (Babson and Feldner, 2010; Milanak et al., 2019; Zhang et al., 2019). Further research is warranted to better contextualize these associations in light of the unique developmental processes of adolescence.

CSA, as basically any other form of adverse childhood experience, is often experienced in co-occurrence with one or many other forms of trauma exposure (Hughes et al., 2017). To isolate the specific effect of CSA, other forms of trauma exposure need to be taken into account. Finally, most studies in this field have relied on cross-sectional designs and reviews have highlighted the need to better document the role of sex in the association between trauma and sleep disturbances (e.g., Kajeepeta et al., 2015; Langevin et al., 2022). Girls are more at risk of experiencing CSA and being diagnosed with PTSD (Haag et al., 2020; Hébert et al., 2019). Sex/gender differences have also been observed regarding sleep disturbances in youth, with girls generally reporting more sleep problems, such as sleep onset difficulties and nightmares (Forest et al., 2022; Wan et al., 2020).

To mitigate the limitations of past studies, this study aimed to: (1) compare the levels of sleep disturbances between youth with and without a history of CSA and with respect to sex, and (2) investigate the mediating role of PTSD symptoms in the association between CSA and sleep disturbances, while controlling for sex, age, and cumulative trauma.

## 2 Methods

### 2.1 Sample and participants

Data were drawn from the *Youth Romantic Relationships Survey* (YRRS). A total of 34 high schools in Quebec, Canada were selected to participate in the study using a one-stage stratified cluster sampling method. All students from grades 9 to 11 were invited to complete the survey at each selected school (response rate of 100% for 320 out of 329 classes; the response rate ranged from 90 to 98% for the remaining classes). Students completed the survey in class twice, the first time in the fall of 2011 (T1) and the second time approximately 6 months later (T2). A total of 8,194 adolescents participated in the study at T1. Because the present study focuses on the role of PTSD symptoms in the association between CSA and sleep disturbances, only adolescents who reported at least one childhood adverse experience at T1 on the *Short form of the Early Trauma Inventory Self-Report* (Bremner et al., 2007) were selected for the analyses (85% of the sample, *n* = 6,848). Of these, 4,993 completed the questionnaire at T2 (73% response rate). Eight adolescents were excluded from the final sample due to invalid responses. The final sample thus included 6,840 adolescents (58% girls, mean age = 15.5 years). Students agreed to participate on a voluntary basis and signed a written consent form. The institutional review board of the affiliated university approved this study.

### 2.2 Measures

#### 2.2.1 Adverse childhood experiences

The Short Form of the Early Trauma Inventory Self-Report (Bremner et al., 2007) was used to screen participants who had been exposed to eight different adverse events, including, among others, the death or serious illness of a close relative, intrafamilial physical abuse, witnessing family violence, and parental divorce or separation. Participants who reported none of the events listed in this measure were excluded from the study.

#### 2.2.2 Child sexual abuse

Two items adapted from Finkelhor et al. (1990) assessed unwanted sexual touching and unwanted sexual intercourse: *“Have you ever been touched sexually when you did not want to, or have you ever been manipulated, blackmailed, or physically forced to touch sexually?”* and *“Has anyone ever used manipulation, blackmail, or physical force, to force or obligate you to have sex (including all sexual activities involving oral, vaginal or anal penetration)?”* A dichotomized score was created based on the presence (1) or absence (0) of any sexual abuse.

#### 2.2.3 Cumulative childhood trauma

Other forms of child maltreatment were assessed using various indicators. Physical abuse and exposure to physical violence were measured using two items from the Early Trauma Inventory Self-Report–Short Form (Bremner et al., 2007): “Have you ever been physically hit by a member of your family?” and “Have you ever witnessed violence against someone, including a member of your family?” Emotional abuse in childhood was evaluated using two items from the Inventory of Parent and Peer Attachment (Armsden and Greenberg, 1987): “My mother tells me hurtful and/or insulting things” and “My father tells me hurtful and/or insulting things.” Exposure to interparental violence was assessed using an adapted version of the Revised Conflict Tactics Scales (CTS2; Straus et al., 1996), consisting of eight items. These items captured the frequency of exposure to interparental psychological (e.g., “insult, swear, shout, yell”) and physical violence (e.g., “push, shove, slap, throw something that could hurt”), with participants indicating whether they had ever witnessed their mother or father engage in these behaviors. Each indicator was dichotomized, and a cumulative trauma score ranging from 0 to 4 was computed to reflect the number of different maltreatment types experienced.

#### 2.2.4 Post-traumatic stress disorder (PTSD)

Nine items from the Abbreviated University of California at Los Angeles PTSD Reaction Index were used to assess PTSD symptoms following sexual abuse or other adverse life experiences listed in the questionnaire (Cohen et al., 2010). The core dimensions of PTSD were assessed, including intrusion symptoms, avoidance, alterations in arousal and reactivity, and negative alterations in cognitions and mood. We removed two items related to sleep difficulties (i.e., nightmares and difficulty falling/staying asleep) to avoid redundancy with our sleep disturbance measure. Adolescents were asked to rate “How much of the time during the past month” they experienced each symptom on a 5-point Likert scale ranging from None of the time (0) to Most of the time (4). The scale showed high internal consistency in the current study (α = 0.86).

#### 2.2.5 Sleep disturbance

Four items were used to assess sleep disturbance at T2. Respondents rated on a 5-point Likert scale ranging from Never (0) to Very often (4) how often they experienced each sleep disturbance over the last 6 months. Items were as follows: “In the last 6 months…” (1) “*Did you have trouble falling asleep or staying asleep?*”; (2) “*Did you have the feeling of not being rested in the morning?*”; (3) “*Did you have the feeling of not getting as much sleep as needed?*,” and (4) “*Did you have nightmares?*” (Noll et al., 2006). A cumulative score ranging from 0 to 16 was calculated. The scale demonstrated high internal consistency (α = 0.82) in the present study.

### 2.3 Statistical analyses

We calculated the prevalence of CSA based on sex using the Pearson *χ*^2^ statistic. We also performed two-way ANOVAs to test the effects of sex, CSA, and the CSA x sex interaction on separate sleep disturbance items, PTSD, and the global score of sleep disturbances. We then conducted a mediation model to estimate the direct and indirect effects of CSA on sleep disturbances with PTSD as a mediator. S*ex*, age, and cumulative trauma were included as covariates in the mediation model. Little’s MCAR test was used to explore the pattern of missingness in the data, and revealed that the data were not MCAR (*χ*^2^ = 236.30, *df* = 9, *p* < 0.001). Results of logistic regressions found that the observed variables predicted missingness, which suggests that a missing at random (MAR) hypothesis is plausible. Hence, missing values were handled using Full Information Maximum Likelihood (FIML). The mediation model was tested using M*plus* software version 8.3 (Muthén and Muthén, 2015). Indirect effects were tested with the ML estimator with the 95% confidence interval bootstrapping technique.

## 3 Results

### 3.1 Descriptive analyses

Overall, 10.4% of our youth sample reported having experienced CSA. A higher prevalence of CSA was observed for girls (15.3%) than boys (3.9%; *χ*^2^ = 276.41, *p* < 0.001). ANOVAs showed that scores on all four sleep disturbance items differed statistically by both sex and CSA status (see Table 1). Girls reported significantly higher mean scores for sleep disturbance and PTSD symptoms than boys. Adolescents with a history of CSA also reported more sleep disturbances and PTSD symptoms than those who did not report a history of CSA. The interaction between sex and CSA did not have a significant effect on the study variables. Pearson’s correlations (Table 2) revealed a significant associations between the independent variable (CSA history) and the mediator (PTSD symptoms), as well as between the mediator and the dependent variable (sleep disturbances), thereby meeting the prerequisites for testing a mediation model.

**Table 1:** ANOVAs results and descriptive statistics for sleep disturbance and PTSD symptoms by sex and sexual abuse history.

Items	*M* (*SD*)				*F*	*p*-value
	CSA	no CSA	Girls	Boys		
Did you have trouble falling asleep or staying asleep? (0–4)	2.36 (1.30)	1.77 (1.33)	2.07 (1.31)	1.48 (1.30)		
*Sex*					45.82	<0.001
CSA					29.61	<0.001
*Sex* x CSA					0.02	0.89
Did you have the feeling of not being rested in the morning? (0–4)	2.80 (1.25)	2.22 (1.35)	2.49 (1.29)	1.98 (1.40)		
*Sex*					32.54	<0.001
CSA					29.42	<0.001
*Sex* x CSA					0.01	0.91
Did you have the feeling of not getting as much sleep as needed? (0–4)	2.99 (1.17)	2.43 (1.32)	2.70 (1.22)	2.16 (1.39)		
*Sex*					35.90	<0.001
CSA					21.53	<0.001
*Sex* x CSA					0.06	0.80
Did you have nightmares? (0–4)	1.63 (1.33)	1.02 (1.90)	1.36 (1.17)	0.66 (0.93)		
*Sex*					99.08	<0.001
CSA					40.61	<0.001
*Sex* x CSA					0.09	0.76
Sleep disturbance - Total (0–16)	9.79 (3.98)	7.44 (4.08)	8.62 (4.00)	6.29 (3.95)		
*Sex*					76.87	<0.001
CSA					51.10	<0.001
*Sex* x CSA					0.08	0.93
PTSD symptoms (0–28)	11.83 (6.99)	7.08 (6.29)	9.22 (6.70)	5.55 (5.83)		
*Sex*					114.97	<0.001
CSA					137.02	<0.001
*Sex* x CSA					1.26	0.26

CSA, Child sexual abuse; PTSD, Post-Traumatic Stress Disorder; M, Mean; SD, Standard Deviation.

**Table 2:** Pearson correlations between study variables.

Variables	1.	2.	3.	4.	5.	6.
Sex (1 = Girls)	–	−0.01	0.11***	0.19***	0.27***	0.28***
Age		–	0.08***	0.06***	0.02	0.01
Cumulative trauma (0–4)			–	0.19***	0.31***	0.27***
CSA (1 = Yes)				–	0.26***	0.18***
PTSD symptoms (0–28)					–	0.36***
Sleep disturbances (0–16)						–

CSA, Child sexual abuse; PTSD, Post-Traumatic Stress Disorder.

***p < 0.001.

### 3.2 Mediation model results

**Figure 1:**
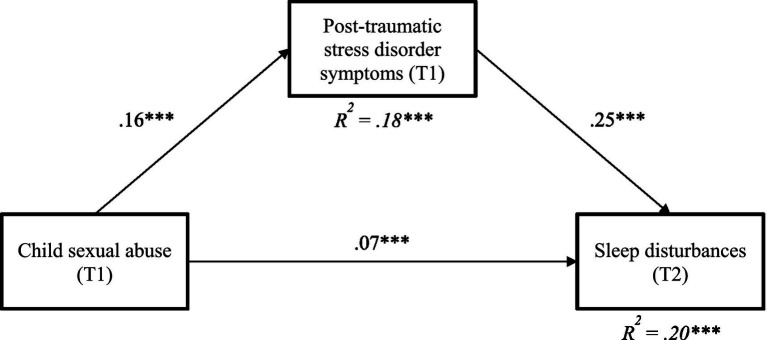
Results of mediation model linking child sexual abuse, post-traumatic stress disorde symptoms and sleep disturbances. Only significant paths are represented. Sex, cumulative trauma, and age included as controlling variables not depicted in the figure for parcimony.

A mediation analysis was conducted to examine whether PTSD symptoms mediated the association between CSA and sleep disturbances after controlling for covariates (sex, cumulative trauma, and age). The indirect effect between CSA and sleep disturbances through PTSD symptoms was significant [*β* = 0.04, 95% bootstrap CI (0.03, 0.05)]. CSA was thus associated with higher levels of PTSD symptoms at T1 [*β* = 0.16, 95% bootstrap CI (0.13, 0.18)], which, in turn, predicted greater sleep disturbances at T2 [*β* = 0.25, 95% bootstrap CI (0.21, 0.28)]. The direct effect between CSA and sleep disturbances remained significant [*β* = 0.07, 95% bootstrap CI (0.04, 0.10)]. The overall model accounted for 20% of explained variance for sleep disturbances and 18% of explained variance for PTSD symptoms. Model fit statistics are not reported as the model was just identified (zero degrees of freedom). Mediation model results are presented in Figure 1.

## 4 Discussion

This study aimed to document sleep disturbances among adolescents with a history of CSA and to assess the role of PTSD symptoms in the association between CSA and sleep disturbances. The results indicate that adolescents with a history of CSA are more likely to experience sleep disturbances than those with other childhood adverse experiences. Moreover, CSA was related to sleep disturbances through PTSD symptoms. Possible explanations for these findings include the impact of PTSD-related hyperarousal and fear responses on sleep onset and sleep quality (Giannakopoulos and Kolaitis, 2021). Reporting a history of CSA also predicted sleep disturbances independently of PTSD symptoms, indicating that other mechanisms could be at play (e.g., Giannakopoulos and Kolaitis, 2021). The indirect association between child sexual abuse and sleep disturbances via PTSD symptoms was significant after controlling for other forms of child maltreatment experienced.

This study has several strengths, including its reliance on a longitudinal design to explore potential mechanisms underlying the link between CSA and sleep disturbances. However, the findings are subject to important limitations. First, this study relied on abbreviated measures of PTSD symptoms as well as of sleep disturbances. Future studies should employ more comprehensive assessments instruments to ensure a more thorough and inclusive evaluation of these constructs. For example, future studies assessing self-reported sleep disturbances in relation to CSA could include more comprehensive sleep assessment instruments such as the Pittsburgh Sleep Quality Index (Buysse et al., 1989), a validated questionnaire assessing several dimensions of self-reported sleep problems within the previous month, including subjective sleep quality, sleep latency, sleep duration, habitual sleep efficiency, sleep disturbances, use of sleeping medication, and daytime dysfunction. Second, the study’s design did not allow for the assessment of bidirectional relationships between PTSD symptoms and sleep disturbances. Further investigations are needed to explore the possible alternative temporal sequences of these variables. The 6-month follow-up interval may not fully capture the longer-term unfolding of mediation processes. In addition, while PTSD symptoms were modeled as a mediator, they could alternatively be conceptualized as covariates or co-occurring responses to trauma, and future longitudinal studies with multiple assessment points would help further disentangle these potential pathways. Finally, the developmental context of adolescence, characterized by biological changes in sleep–wake cycles, increased emotional reactivity, and evolving social demands, may further exacerbate the impact of trauma on sleep disturbances. Future studies would benefit from explicitly examining how developmental stages interact with trauma-related variables in predicting sleep outcomes.

Our results offer several implications for clinical practice. Given the association between PTSD and sleep disturbances in teenagers with a history of CSA, therapeutic interventions should integrate PTSD treatment with strategies specifically targeting sleep disturbances. An approach combining evidence-based practices tailored to address both PTSD (e.g., Trauma-Focused Cognitive Behavioral Therapy; Cohen et al., 2016) and its impact on sleep and persistent nightmares (e.g., imagery rehearsal therapy, cognitive behavioral therapy for insomnia; Miller et al., 2020) could be warranted.

## 5 Conclusion

This longitudinal study highlights the significant impact of CSA on sleep disturbances among adolescents, with PTSD symptoms playing a crucial mediating role. The findings underscore the necessity for practitioners to routinely assess sleep disturbances in adolescents with a history of CSA and to integrate PTSD-focused therapies with sleep-specific interventions.

## Data Availability

The raw data supporting the conclusions of this article will be made available by the authors, without undue reservation.
